# *Tremella fuciformis* Polysaccharide Induces Apoptosis of B16 Melanoma Cells via Promoting the M1 Polarization of Macrophages

**DOI:** 10.3390/molecules28104018

**Published:** 2023-05-11

**Authors:** Lingna Xie, Guangrong Liu, Zebin Huang, Zhenyuan Zhu, Kaiye Yang, Yiheng Liang, Yani Xu, Lanyue Zhang, Zhiyun Du

**Affiliations:** 1 School of Biomedical and Pharmaceutical Sciences, Guangdong University of Technology, Guangzhou 510006, China; 1112012007@mail2.gdut.edu.cn (L.X.); 1112012002@mail2.gdut.edu.cn (Z.H.); 2112012033@mail2.gdut.edu.cn (Y.L.); 2111906011@mail2.gdut.edu.cn (Y.X.); 2 Infinitus Company Ltd., 11 Sicheng Road, Tianhe District, Guangzhou 510000, China; jim.liu@infinitus-int.com (G.L.); kyle.yang@infinitus-int.com (K.Y.); 3 State Key Laboratory of Food Nutrition and Safety, Tianjin University of Science and Technology, Tianjin 300457, China; zhyuanzhu@tust.edu.cn; 4 College of Food Science and Engineering, Tianjin University of Science and Technology, Tianjin 300457, China; 5 Guangdong Provincial Key Laboratory of Plant Resources Biorefinery, Guangdong University of Technology, Guangzhou 510006, China

**Keywords:** *Tremella fuciformis* polysaccharide, immunoregulation, co-culture, macrophages, B16

## Abstract

Anti-tumor activity of *Tremella fuciformis* polysaccharides (TFPS) has been widely reported, but its mechanism remains poorly understood. In this study, we established an in vitro co-culture system (B16 melanoma cells and RAW 264.7 macrophage-like cells) to explore the potential anti-tumor mechanism of TFPS. Based on our results, TFPS exhibited no inhibition on the cell viability of B16 cells. However, significant apoptosis was observed when B16 cells were co-cultured with TFPS-treated RAW 264.7 cells. We further found that mRNA levels of M1 macrophage markers including iNOS and CD80 were significantly upregulated in TFPS-treated RAW 264.7 cells, while M2 macrophage markers such as Arg-1 and CD 206 remained unchanged. Besides, the migration, phagocytosis, production of inflammatory mediators (NO, IL-6 and TNF-α), and protein expression of iNOS and COX-2 were markedly enhanced in TFPS-treated RAW 264.7 cells. Network pharmacology analysis indicated that MAPK and NF-κB signaling pathways may be involved in M1 polarization of macrophages, and this hypothesis was verified by Western blot. In conclusion, our research demonstrated that TFPS induced apoptosis of melanoma cells by promoting M1 polarization of macrophages, and suggested TFPS may be applied as an immunomodulatory for cancer therapy.

## 1. Introduction

Cancer is a major cause of death, and its treatment is a hot topic worldwide. Traditional treatment methods for cancer (surgery, chemotherapy, and radiotherapy) exhibit obvious efficacy, but these methods may cause serious side effects or increase tumour recurrence risk [1,2]. As a new cancer treatment strategy, immunotherapy enhances the antitumour immune response by reactivating or strengthening the immune system, which possesses promising therapeutic potential [3]. Recently, immunotherapies such as chimeric antigen receptor-engineered T cells (CAR-T) and immune checkpoint inhibitors showed encouraging results in the clinic [4]. However, most immunotherapies are T-cell-dependent, and some patients do not benefit from them. Therefore, the use of the innate immune system to control tumors provides new possibilities for the development of next-generation immunotherapy [5].

Macrophages are innate immune system components and play a pivotal role in the tumor microenvironment (TME). Macrophage cells infiltrating tumor tissues are known as tumor-associated macrophages (TAMs) [6]. TAMs are involved in tumor immune escape, angiogenesis, growth, and transfers [7]. Macrophages can be polarised into two main phenotypes under the stimulation of different environmental factors: immune activation (i.e., M1-like) and immunosuppressive (i.e., M2-like) phenotypes [8]. The cluster of differentiation (CD) 80 is considered a characteristic surface molecule of M1 macrophages, whereas CD206 is a surface marker of M2 macrophages [9]. Generally, M1 macrophages are activated by bacterial lipopolysaccharide (LPS) and interferon-γ (IFN-γ) produced by T helper 1 (TH1) cells, while M2 macrophages are activated by cytokines produced by T helper 2 (TH2), such as IL-4 and IL-13. M1 macrophages produce ROS, nitric oxide (NO), and pro-inflammatory cytokines, such as interleukin-6 (IL-6), IL-12, and tumor necrosis factor-α (TNF-α), which participate in the inflammatory response and suppress cancer. In contrast, M2 macrophages exert anti-inflammatory effects by producing immunosuppressive molecules such as transforming growth factor-β (TGF-β) and IL-10, thereby promoting tumor progression [10]. Most macrophages manifest an M2-like phenotype in TAMs, which indicates that the TME is immunosuppressive and governs tumor occurrence, progression, metastasis, and recurrence [11,12]. Therefore, the polarisation of TAMs into the anti-tumor M1 phenotype gradually became a research hotspot.

In recent years, numerous studies demonstrated the multiple pharmacological activities of polysaccharides obtained from natural sources, particularly their immunomodulatory activity. Various polysaccharides were found to polarise monocytes, macrophages, and TAMs into M1-like macrophages by direct or indirect interactions [13,14,15]. *Tremella fuciformis*, commonly known as snow fungus or silver ear in Chinese, belongs to the family Tremellaceae [16]. It is an edible and highly nutritious fungus that gained popularity in Asia. In terms of nutrition, *Tremella fuciformis* is rich in proteins, polysaccharides, dietary fiber, and various trace elements [17]. *Tremella fuciformis* polysaccharide (TFPS) has various biological activities, including immunomodulatory, anti-tumor, antioxidant, whitening skin, anti-inflammatory, neuroprotective, hypoglycaemic, and hypolipidaemic effects, among which immunomodulatory activity includes activation of macrophages, monocytes, T-lymphocytes, B-lymphocytes, and humoral immunity [18,19]. Additionally, many studies confirmed the protective effect of TFPS in different tumor-bearing mice [20,21,22,23]. Some studies also indicated that TFPS has antitumor activity in vitro [24]. In 2002, the Chinese Food and Drug Administration (SFDA) approved a TFPS-related drug, Tremella polysaccharide enteric-coated capsules, to treat leukopenia caused by chemotherapy or radiotherapy [25]. However, the molecular mechanisms of TFPS for immune regulation and anti-tumor activity, as well as the contribution of its immunomodulatory activity to the anti-tumor effect, are still poorly understood. Thus, this work sought to gain new insights into the role of TFPS in tumor suppression by regulating macrophage M1 polarization. In this study, we verified the anti-tumor effect of TFPS in a co-culture system (B16 cells and RAW 264.7) and further explored the mechanism underlying the induction of macrophage polarisation to the M1 phenotype.

## 2. Results

### 2.1. Extraction and Characterization of TFPS

**Figure 1:**
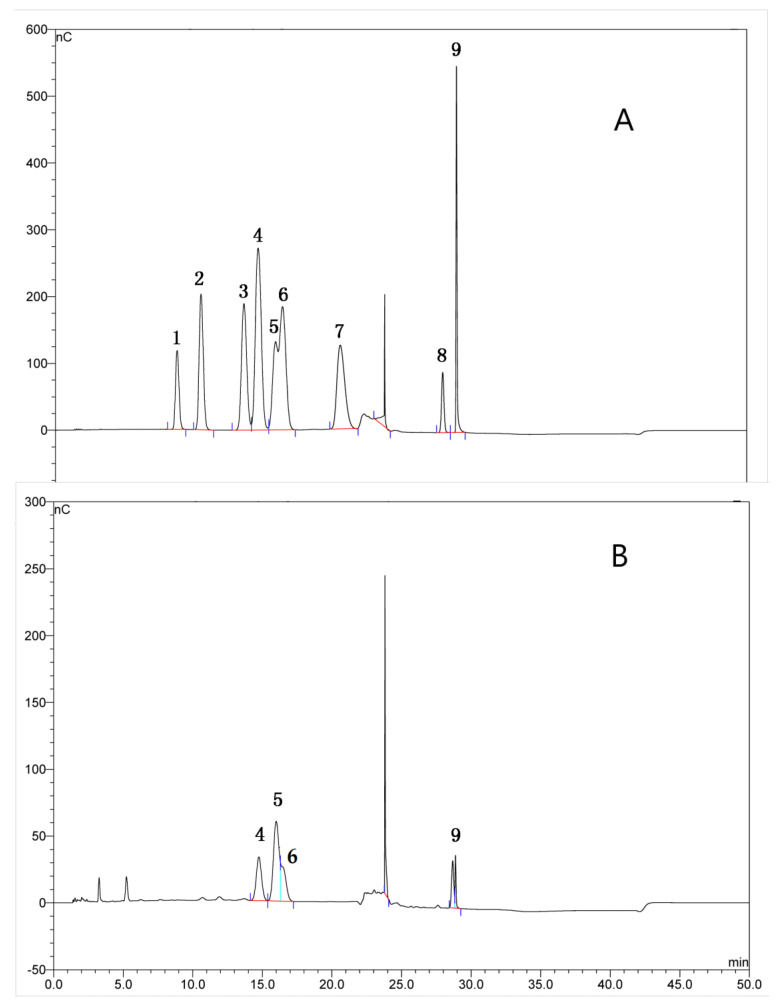
The monosaccharide composition analysis of TFPS from *Tremella fuciformis*. The chromatogram of mixed standard monosaccharides (**A**), and the chromatogram of TFPS after hydrolysis (**B**). 1-Rhamnose, 2-L-Arabinose, 3-Galactose, 4-Glucose, 5-Xylose, 6-Mannose, 7-Ribose, 8-D-Galacturonic acid and 9-Glucuronic acid in (**A**).

The total sugar content of TFPS, determined by the phenol sulphuric acid method, was 78.28%. The monosaccharide composition of TFPS after hydrolysis by trifluoroacetic acid was determined by high performance liquid chromatography. The results showed that TFPS consisted of xylose, glucose, mannose, and glucuronic acid in a molar ratio of 3.7:1.9:1.5:1 (Figure 1A). TFPS was determined by high performance gel permeation chromatography (HPGPC), revealing a molecular weight range of 32 to 918 kDa, mainly polysaccharides with a molecular weight of 918 kDa, accounting for 63%. A recent study indicated that alkali-extracted yeast phase polysaccharides from *Tremella fuciformis* comprised different xylose, mannose, and glucose ratios [26]. TFPB is a fermented *Tremella fuciformis* polysaccharide whose monosaccharide components are mannose, glucuronic acid, glucose, galactose, xylose, and rhamnose [27]. The monosaccharide composition of TFPS was similar to that of fermented *Tremella fuciformis* polysaccharides, but the composition of fermented *Tremella fuciformis* polysaccharides was more complex.

### 2.2. Extraction and Characterization of TFPS

**Figure 2:**
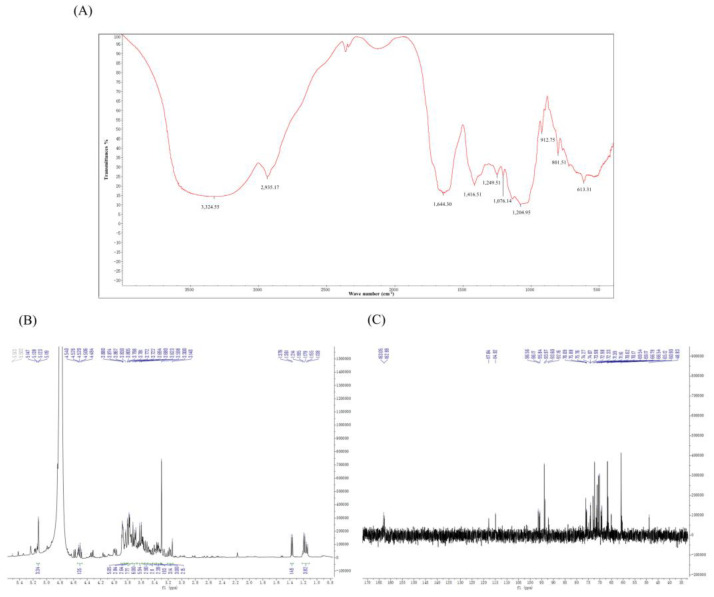
The infrared spectrum and NMR spectra of TFPS. FT-R spectrum (**A**), ^1^H NMR (**B**), and ^13^C NMR (**C**).

The functional groups of TFPS corresponding to the absorption peaks were speculated based on FTIR characterization. The infrared absorption spectrum of TFPS is shown in Figure 2A. The vibration region at 3414 cm^−1^ corresponded to the O-H stretching vibration, and the absorption peak at 2932 cm^−1^ corresponded to the C-H stretching vibration. The absorption peak in this region was characteristic of sugar. The absorption peak at 1603 cm^−1^ corresponded to the skeletal vibrations of the aromatic and heterocyclic rings. The absorption peaks at 1409 cm^−1^ and 1249 cm^−1^ corresponded to the absorption peaks caused by the CH variable-angle vibration. The strong peak at 1076 cm^−1^ may be attributed to the CO stretching vibration, and the characteristic peak at 801 cm^−1^ indicated the existence of an α-glycosidic bond. Infrared spectroscopy showed that TFPS contained the typical functional groups of polysaccharides.

There were narrow regions in the 1H-NMR spectrum of TFPS ranging from 3.0 to 5.5 ppm (^1^H NMR) and 60 to 110 ppm (^13^C NMR) that were typical of polysaccharides. As shown in Figure 2B, the peaks at δ5.0–5.5 ppm were considered to be α-isomeric protons and the β-isomeric proton signals were located at δ4.2–4.7 ppm, respectively. Most of the proton signals in the 1H spectra of polysaccharides were concentrated in the region of δ3.0–4.0 ppm, with significant overlap. However, due to the complex nature of TFPS, they were not well separated. The heterotopic carbon with a chemical shift of δ90–103 ppm in ^13^C NMR is an α-isomeric proton. As shown in Figure 2C, a weak signal at the low field of δ160–180 ppm indicates that the TFPS may contain glyoxylate.

### 2.3. Effect of TFPS Treatment on B16 Cells and RAW 264.7 Cells in Co-Culture System

**Figure 3:**
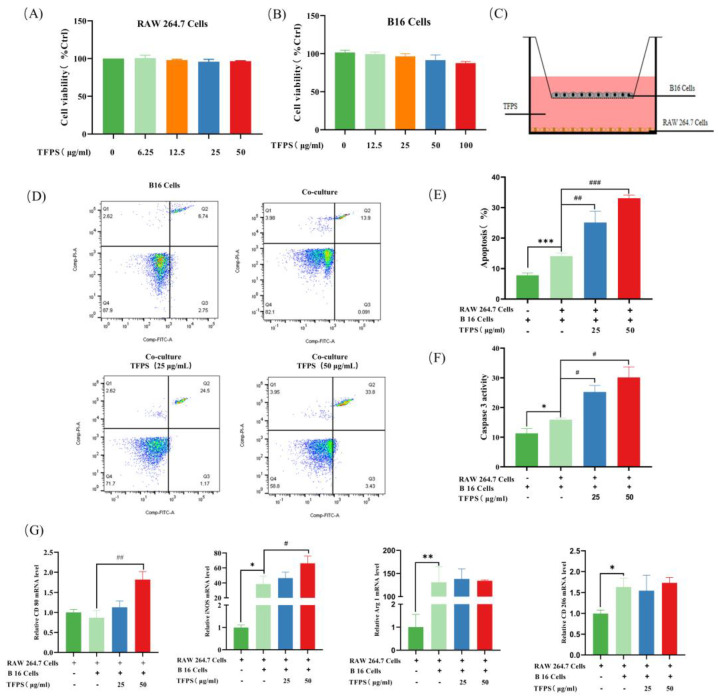
TFPS induced polarisation of RAW 264.7 cells and apoptosis of B16 cells. RAW 264.7 cells (5 × 10^3^ cells/well) and B16 cells (5 × 10^3^ cells/well) were treated with different TFPS doses (1–50 μg/mL) for 24 h, respectively, and the cell viability (**A**,**B**) was determined by MTT method. (**C**) Schematic diagram of the co-culture system. (**D**,**E**) Flow cytometry was used to detect the apoptosis of B16 cells. Four groups were there in this experiment, including blank group (only B16 cells), co-cultured group (B16 cells co-cultured with RAW 264.7 cells), 25 and 50 μg/mL of TFPS-treated (B16 cells co-cultured with RAW 264.7 cells) groups. (**F**) Detection of active Caspase-3. (**G**) The iNOS, Arg 1, CD80, and CD206 mRNA expressions in RAW 264.7 cells in the co-culture system. Values were shown as means ± SEM (n = 3). * *p* < 0.05, ** *p* < 0.01, *** *p* < 0.001, compared with the blank group. # *p* < 0.05, ## *p* < 0.05, ### *p* < 0.001, compared with the co-cultured group.

We first determined the effect of TFPS on the survival of B16 cells and RAW 264.7 cells by MTT assay. The results showed that TFPS showed no significant change in the survival rate of B16 cells and RAW 264.7 cells below 50 µg/mL (Figure 3A,B). Next, we explored the effect of TFPS treatment on RAW 264.7 cells and B16 cells in a co-culture system. As shown in Figure 3C, B16 cells in transwell inserts were co-cultured with RAW 264.7 cells, and both cells could share a medium. B16 cells were stained with annexin V-PI and the apoptosis rate was analysed by flow cytometry. Interestingly, compared with the control group, the apoptosis rates of B16 cells in the 25 and 50 μg/mL TFPS-treated groups were significantly increased (*p* < 0.01) in a concentration-dependent manner, by 1.76 and 2.43-fold, respectively (Figure 3D,E). Furthermore, the activity of caspase-3 was significantly increased in B16 cells after the 25 and 50 μg/mL TFPS treatment (Figure 3F).

It could be observed that both M1 and M2 polarisation phenotypes of RAW 264.7 were significantly increased in the co-culture system. In the control group, both the mRNA levels of the M1 macrophage marker (iNOS) and M2 macrophage marker (Arg1 and CD206) were upregulated in RAW 264.7, of which iNOS and Arg1 were statistically significant (Figure 3G). A previous study showed that resting state macrophages (M0) can be polarised into mixed M1 and M2 phenotypes upon the induction of melanoma exosomes, and this result agreed with our observation [28]. Compared to the control group, the iNOS and CD80 mRNA levels in the 50 μg/mL TFPS-treated group were significantly up-regulated by 1.72 and 2.10-fold, respectively. However, the mRNA expression of M2 macrophage markers, such as Arg1 and CD206, was essentially unchanged. Based on these findings, TFPS may promote apoptosis of B16 cells by polarising TAMs to an antitumor M1 phenotype, thereby exerting anti-tumor activity.

### 2.4. TFPS Enhanced the Migration and Phagocytosis of RAW 264.7 Cells

**Figure 4:**
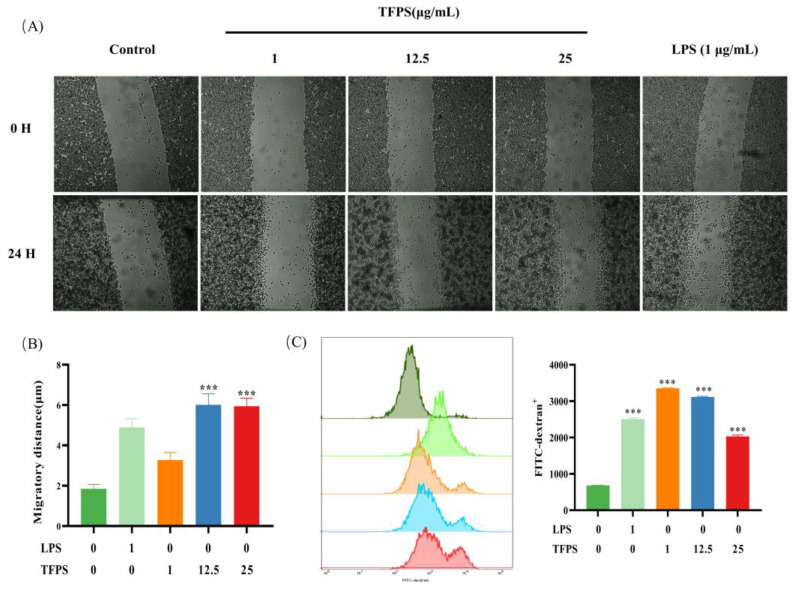
The effect of TFPS on the migration and phagocytosis of RAW 264.7 cells. (**A**) Cell migration images in the indicated concentration of TFPS or LPS induced RAW 264.7 cells were collected at 0 h and 24 h. (**B**) The migration distance. (**C**) The phagocytosis of FITC-dextran in RAW 264.7 cells induced by the indicated concentration of TFPS or LPS was analysed by flow cytometry. Values are shown as mean ± SEM (n = 3). *** *p* < 0.001, compared with the untreated group.

Since macrophage infiltration into tumor sites is a prerequisite for macrophages to inhibit tumor progression, we explored the effect of TFPS on the migratory ability of RAW 264.7. It could be observed that the migration ability of RAW 264.7 cells was significantly improved after stimulation with LPS or 1, 12.5, or 25 μg/mL of TFPS (Figure 4A). The migration distance increased by 2.59-, 1.82-, 3.19-, and 3.34-fold (Figure 4B). We also detected the effect of LPS or different concentrations of TFPS (1, 12.5, and 25 μg/mL) on the phagocytic activity of RAW 264.7 cells by using FITC-labelled dextran. The flow cytometry results showed that both LPS and TFPS enhanced the phagocytic ability of RAW 264.7 cells (Figure 4C), and TFPS exhibited a concentration-dependent trend. Macrophage migration and phagocytosis are important for tumor immune surveillance [29]. Our findings demonstrate that TFPS can promote phagocytosis and migration of macrophages, indicating that TFPS has an immunomodulatory function.

### 2.5. Effect of TFPS on the Production of Pro-Inflammatory Mediators

**Figure 5:**
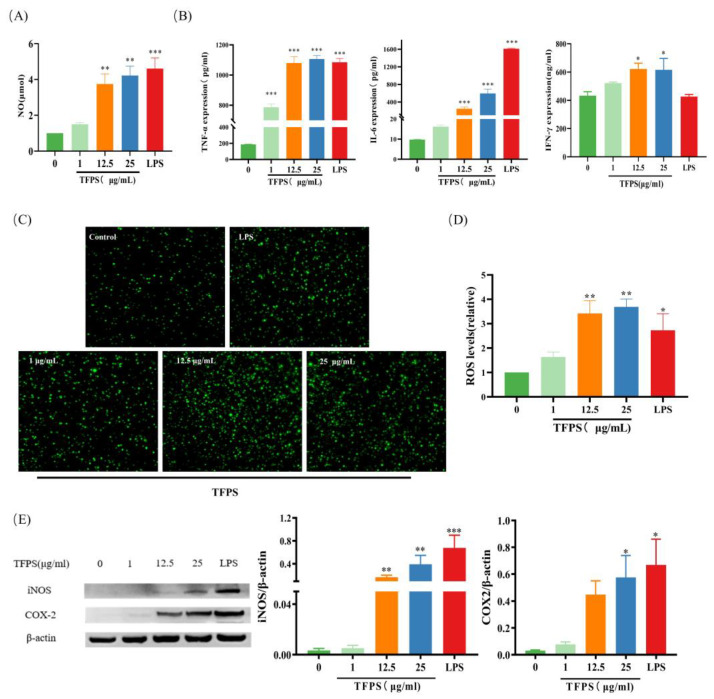
TFPS stimulated the production of pro-inflammatory mediators. RAW 264.7 cells (2 × 10^5^ cells/well) were treated with the indicated concentration of TFPS or LPS for 24 h. NO (**A**), TNF-α and IL-6 (**B**) from supernate were detected. ROS were determined by staining with DCFH-DA and observed by confocal microscopy (40×) (**C**) and the relative ROS levels compared with the control (**D**). (**E**) The iNOS and COX-2 expressions were determined by Western blot and β-actin was used as a control. Values are expressed as means ± SEM (n = 3). * *p* < 0.05, ** *p* < 0.01, *** *p* < 0.001, compared with the untreated group.

As the production of pro-inflammatory mediators is a hallmark of M1 macrophages, we determined the effect of TFPS on the production of several pro-inflammatory mediators in RAW 264.7 cells. After treatment with different doses (1, 12.5, and 25 μg/mL) of TFPS for 24 h, the NO, TNF-α, IL-6, and IFN-γ levels in the supernatant were directly measured. Drastic NO, TNF-α, IL-6, and IFN-γ content upregulation was observed in a dose-dependent manner (Figure 5A,B). As a secondary inflammation messenger, ROS are upregulated during the inflammatory response [30]. Compared with the untreated group, ROS levels in RAW 264.7 cells were increased after treatment with different concentrations of TFPS (1, 12.5, and 25 μg/mL) or LPS (Figure 5C,D). NO secretion requires iNOS processing, which is thought to contribute to COX-2 production. Therefore, the iNOS and COX-2 protein levels were determined. As shown in Figure 5E, different TFPS concentrations promoted iNOS and COX-2. These results suggested that TFPS effectively promoted the polarisation of RAW 264.7 macrophages to the M1 phenotype.

### 2.6. Potential Targets and Signalling Pathways of TFPS Affecting TAMs

**Figure 6:**
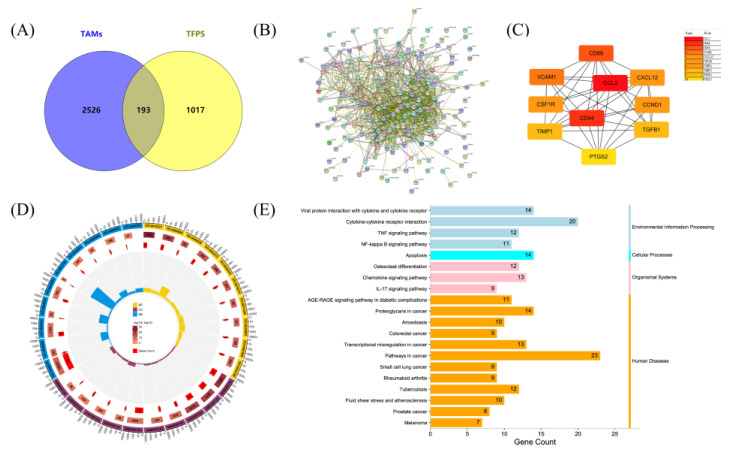
Network pharmacology analysis revealed possible targets and molecular mechanisms by which TFPS regulated the polarisation of TAMs. (**A**) Venn diagram showed the target interaction between TAMs and TFPS. PPI network of 193 intersected targets (**B**) was generated by STRING, and the top 20 targets (**C**) were presented. GO terms (**D**) and KEGG pathway (**E**) of 193 intersected targets were visualized.

Previous data demonstrated that RAW264.7 macrophages would be polarised by TFPS to an M1 phenotype, which was favourable for tumour suppression in the TME. To explore the potential targets and signalling pathways of TFPS in TAMs, we performed a network pharmacology analysis. A total of 2719 potential TAM targets were collected from the GeneCards database. After merging the targets in the TCMSP, HIT, and HERB databases, 1210 putative TFPS targets were identified. Ultimately, 193 potential targets were obtained by intersecting the targets of TFPS with those of TAMs (Figure 6A). These targets were imported into STRING to generate the PPI network. The PPI network contained 193 nodes and 1230 edges, with an average node degree of 12.7 (Figure 6B). Ten hub targets with the highest degree was displayed using Cytoscape: CCL2, CD44, CD68, VCAM1, CXCL12, CSF1R, CCND1, TIMP1, TGFB1, and PTGS2 (Figure 6C).

We further performed GO classification and KEGG pathway enrichment analysis of the 193 intersecting targets. The results showed that 289, 51, and 51 terms were enriched in BP, CC, and MF, respectively (*p* < 0.05). The top 10 BP, CC, and MF are displayed. As shown in Figure 6D, targets regulated by TFPS in TAMs were mainly enriched in the extracellular space (GO:0005615), extracellular region (GO:0005576), and cell surface (GO:0009986). These targets were involved in biological processes such as cytokine-mediated signaling pathway (GO:0019221), positive cell migration regulation (GO:0030335), and positive ERK1 and ERK2 cascade regulation (GO:0070374). Additionally, their molecular functions included protein binding (GO:0005515), chemokine activity (GO:0008009), and protein homodimerization activity (GO:0042803). Fifty-two human pathways were enriched in the KEGG database (*p* < 0.05). Surprisingly, the intersecting targets were significantly associated with melanoma (Figure 6E), suggesting that TFPS may have a therapeutic effect on melanoma. These targets were also enriched in the TNF, NF-κB, and IL-17 signaling pathways, whereas the NF-κB and MAPK signaling pathways were both included in the TNF and IL-17 signaling pathways (Figure S1A,B). Therefore, the NF-κB and MAPK signaling pathways were selected to verify the regulatory effects of TFPS on macrophages.

### 2.7. TFPS Activated MAPK and NF-κB Signalling Pathways

**Figure 7:**
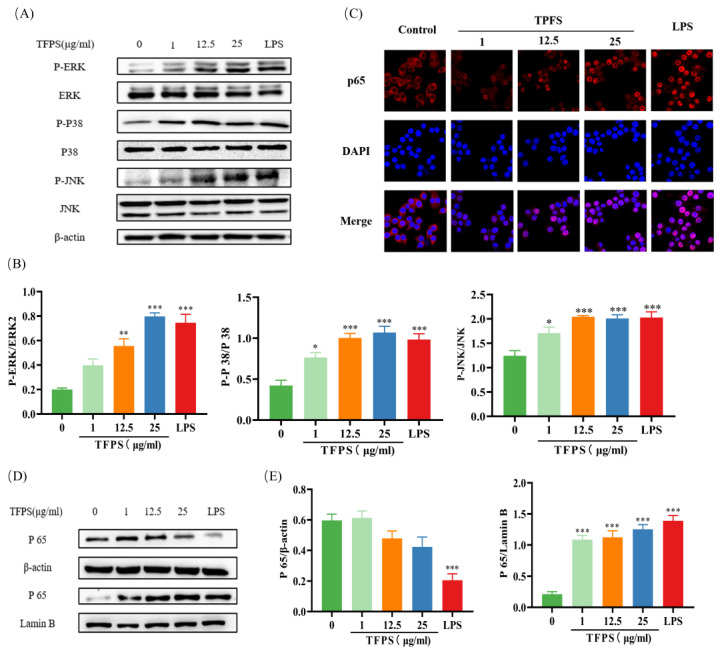
Effect of TFPS on the MAPK and NF-κB signalling pathway. RAW 264.7 cells (5 × 10^5^ cells/mL) were treated with the indicated concentration of TFPS or LPS for 1 h. (**A**,**B**) The expression levels of p-ERK/ERK, p-p38/p38 and p-JNK/JNK were determined by Western blot. (**C**) Immunofluorescence was used to determine the expression levels of NF-κB (p65) in the cytosolic fraction and nuclear fraction. (**D**,**E**) The expression levels of NF-κB (p65) in the cytosolic fraction and nuclear fraction were determined by Western blot. Data are presented as the means ± SEM (n = 3). * *p* < 0.05, ** *p* < 0.01, *** *p* < 0.001, compared with the untreated group.

According to previous data, we selected both NF-κB and MAPK signaling pathways to verify the regulatory effects of TFPS on macrophages. We explored whether MAPKs are involved in regulating the M1-type polarisation of macrophages by TFPS. The ERK1/2, JNK, and p38 phosphorylation levels were significantly increased in LPS-induced RAW 264.7, indicating that the MAPK signaling pathway was activated. Interestingly, the expression levels of p-ERK, p-JNK, and p-p38 proteins were also significantly increased in TFPS-treated RAW 264.7 cells at 1 μg/mL, 12.5 μg/mL, and 25 μg/mL (Figure 7A,B).

After activation of the NF-κB signaling pathway, IκB is phosphorylated by IκB kinase and then dissociated from NF-κB, allowing the NF-κB p65 subunit to translocate into the nucleus. Therefore, an increase in p65 protein content in the nucleus reflects the activation of NF-κB [31]. In this study, we detected the nuclear transcription of p65 using immunofluorescence. Compared to the untreated group, the fluorescence intensity of NF-κB p65 in the nucleus of the TFPS treatment group was significantly increased, indicating that TFPS promoted the transport of p65 from the cytoplasm to the nucleus (Figure 7C). To verify this result further, Western blotting was performed. As shown in Figure 7D,E, the NF-κB (p65) expression level was significantly decreased in the cytosolic fraction and increased in the nuclear fraction in the TFPS-treated groups.

## 3. Discussion

Tumor immune microenvironment (TIME) plays an important role in tumor development [32]. TAMs is the main component of the tumor environment, and there are two phenotypes of macrophages, M1 subtype and M2 subtype. M1 subtype macrophages participate in the pro-inflammatory killing response, secrete proinflammatory cytokines, promote immune response, and enhance tumor killing ability. M2 macrophages play an important role in the immune response by participating in repair, secreting anti-inflammatory cytokines, repairing damaged tissues, promoting angiogenesis, and mediating tumor immune escape [33]. Several recent studies suggested that redirecting TAMs to the M1 phenotype that promotes immune responses to tumors, acting as a tumor immunotherapy, is an attractive therapeutic intervention [34].

Many plant polysaccharides can activate macrophages and enhance immune function with less toxicity and side effects, which attracted more and more attention from researchers. Recent studies showed that *Homogeneous Polyporus* Polysaccharide (HPP) can induce TAMs polarization to the M1 phenotype and inhibit bladder tumors [35]. Guo et al. reported that the combination of *maca* polysaccharide (MPW) and its derivative (C-MPW) and doxorubicin (Dox) had a stronger anti-tumor effect and a synergistic tumor immunotherapy effect. Moreover, TAMs can be redirected to M1 phenotype that promotes tumor immune response through NF-κB, STAT1, and STAT3 pathways [36]. Li et al. found that *Tremella fuciformis* derived polysaccharide (TFP) could participate in anti-tumor effects through lipid metabolism, and demonstrated that high dose of TFP (1250–5000 µg/mL) directly inhibited the proliferation of B16 cells. It promotes B16 cell apoptosis and induces G2/M cell cycle arrest [37]. In our study, low doses of TFPS (12.5–50 µg/mL) had no effect on B16 cell proliferation. Interestingly, in a co-culture model mimicking the tumor microenvironment (B16 cells and RAW 264.7 macrophages), TFPS treatment (12.5–50 µg/mL) significantly enhanced caspase3-dependent apoptosis of B16 cells. This suggests that the antitumor effect of low doses of TFPS may be caused by promoting the M1 polarization of macrophages. The results showed that TFPS promoted the expression of cellular markers iNOS and CD80 associated with M1 phenotype, which proved that TFPS could promote TAMs polarization to M1 phenotype.

The above evidence prompted us to explore the underlying mechanism of TFPS on M1 polarization of macrophages. Using network pharmacology, it was found that the cross-targets of TFPS and TAMs were related to TNF signaling pathway, NF-κB signaling pathway and IL-17 signaling pathway. Some studies showed that polysaccharides can activate macrophages through TLR4, and MAPK is an important downstream signaling molecule mediating macrophage activation and expression of inflammatory factors [38]. The results of Ueno et al. showed that polysaccharide PG activated macrophages via TLR4, and when macrophages were pretreated with specific inhibitors of the three kinases ERK1/2, SAPK/JNK, and p38 MAPK, the activation of polysaccharide PG in macrophages was inhibited [39]. This suggests that the MAPK pathway is involved in the activation of macrophages by PG. Studies found that Ac-PMEP 3 can increase the expression of p-p38 in normal macrophages to mediate macrophage activation, while Pachymia coporia can stimulate macrophages by activating NF-κB and p38 kinase [40,41]. The results of the present study showed that TFPS induced the activation of MAPK and NF-κB pathways in RAW264.7 macrophages, which may help to explain the mechanism by which TFPS promote M1 polarization. However, Lee et al. showed that TFPS inhibited iNOS/NO and COX-2/PGE 2 production induced by LPS in RAW 264.7 cells [42]. Subsequently, Ruan et al. found that TFPS reduced LPS-induced upregulation of miR-155 and NF-κB activation in RAW 264.7 cells [43]. These differences in results may be related to the origin, extraction process, composition structure, and TFPS concentration.

## 4. Materials and Methods

### 4.1. Materials and Chemicals

The lipopolysaccharide (LPS) and dimethyl sulfoxide (DMSO) were purchased from Sigma-Aldrich (St. Louis, MO, USA). The 3-(4,5-dimethylthiazol-2-yl)-2,5-diphenyl-tetrazole-bromide (MTT), BCA protein assay kit, and 4′,6-Diamidino-2-phenylindole dihydrochloride (DAPI) were purchased from Beijing Solarbio Science and Technology Co., Ltd. (Shanghai, China). TNF-α ELISA kits (SMK2868A) were provided by Jiangsu Sumeike Biological Technology Co., Ltd. (Jiangsu, China). The nitric oxide (NO) test kit, reactive oxygen species (ROS) detection kit, and COX2 antibody were purchased from Beyotime Biotech. Inc. (Shanghai, China). NE-PER Nuclear and Cytoplasmic Extraction Kit were provided by Thermo Fisher (Waltham, MA, USA). NOS2 antibody (CSB-PA003464) and a secondary anti2212rabbit antibody were bought from Cusabio (Wuhan, China, http://www.cusabio.com). The IL-6 ELISA kit (mouse) was provided by Jiangsu Meibiao Biological Technology Co. Ltd. (Jiangsu, China). SAPK/JNK (9252S), phospho-SAPK/JNK (9251S), p44/42MAPK (9102S), phospho-p44/42MAPK (9101S), p38 (9212S), phospho-p38 (9211S), NF-κB (8242S), phospho-NF-κB (3033S), lamin B1 (12586S), beta-actin (58169S) were obtained from Cell Signaling Technology (Danvers, MA, USA). The 35 mm confocal dish used for immunofluorescence was purchased from NEST Biotechnology (Jiangsu, China). The IFN-γ ELISA kit was purchased from Jiangsu Meimian Industrial Co., Ltd. (Jiangsu, China). FITC-dextran (4 kDa) was purchased from Aladdin (Shanghai, China). Other reagents used in this study were analytical grade.

### 4.2. Preparation of Polysaccharides

The fruit bodies of *Tremella fuciformis* were freeze-dried under a vacuum for 48 h, then ground and passed through a 60-mesh sieve. Powder of *Tremella fuciformis* was extracted with 10-fold hot water [44]. The extractive solution was concentrated at 55 °C using a rotary evaporator, and then precipitated by 4-fold volume of 80% ethanol. The mixture was then heated for 2 h, centrifuged, and filtered (4000 rpm, 15 min, 4 °C). The ethanol was removed by rotary evaporation under reduced pressure, and the crude polysaccharide was obtained by lyophilization for 48 h. To obtain purified polysaccharide, the crude polysaccharide was deproteinized several times by the Sevag method. Finally, TFPS was prepared by collecting, precipitating, and freeze-drying the fractions.

### 4.3. Analysis of Monosaccharide Composition

The total sugar content in TFPS was measured by the phenol sulfuric acid method [45]. Monosaccharide compositions in TFPS were determined by Gas Chromatography-Mass Spectrometer (GC-MS). The TFPS or standard was hydrolyzed with 2 M trifluoroacetic acid (TFA) for 3 h at 110 °C. TFA was removed by blowing with nitrogen after cooling. The hydrolysate was transferred to a 50 mL volumetric flask, before being diluted to 40 ppm and loaded. The chromatographic column is a DionexCarboPacTM PA10 ion exchange column. Elution was performed using phase A (91%, ultrapure water) and phase B (9%, NaOH solution), and the flow rate was 1 mL/min. The column temperature was 30 °C. A pulsed amperometric detector was used for detection. The injection volume was 10 μL and the analysis time was 60 min.

### 4.4. Determination of Molecular Weight

The molecular weight distribution of polysaccharides was measured by high-performance liquid chromatography (HPLC). The analysis conditions of HPLC were described as follows [46]. A TSK-GEL G4000PWxl chromatographic column was used with a column temperature of 30 °C. The injection volume of samples was 20 μL. Ultrapure water was used as the mobile phase at a flow rate of 0.6 mL/min. The differential detector temperature was 35 °C. Standard dextran T-10, T-40, T-70, T-110, T-500, and T-2000 were used as standard products. Finally, the average Mw (average molecular weight) value of the polysaccharide was determined based on the standard curve.

### 4.5. Analysis of Infrared Spectroscopy and Nuclear Magnetic Resonance

In infrared spectroscopy, tablets containing 1 mg of TFPS and 100 mg of potassium bromide were processed as infrared absorption spectra. The scanning range was 400 cm^−1^–4000 cm^−1^, the resolution ratio was 4 cm^−1^ and the scanning times were 16 [47].

In nuclear magnetic resonance, 50 mg TFPS was dissolved in 0.6 mL deuterium oxide (D_2_O). The information on ^1^H-NMR and ^13^C-NMR was collected with an NMR spectrometer (AVIII-400M, Bruker, Switzerland).

### 4.6. Cell Culture

RAW 264.7 macrophage cells and B16F10 melanoma cells were obtained from the ATCC (Rockville, MD, USA). The cells were cultured in DMEM supplemented with 10% fetal bovine serum(FBS)and 1% penicillin-streptomycin in a humidified atmosphere containing 5% carbon dioxide at 37 °C.

### 4.7. Cell Viability Assay

The effect of TFPS on the proliferation of RAW 264.7 cells and melanoma B16 cells was determined by the MTT method [48]. First, the cells were seeded in a 96-well plate at 5 × 10^3^ cells/well and maintained for 24 h. Then, RAW 264.7 cells or B16 cells were treated with TFPS at different concentrations (1, 6.25, 12.5, 25, 50 μg/mL), followed by maintenance for another 24 h. After removing the spent medium, 100 μL of MTT solution (500 μg/mL) was added to each well and incubated at 37 °C for 4 h. Then, the supernatant was discarded and 150 μL of DMSO solution was added to each well. Finally, the absorbance values were detected at 570 nm.

### 4.8. Detection of B16 Cells Apoptosis

B16 cells (7.5 × 10^5^ cells/well) were co-cultured with RAW 264.7 cells (1 × 10^6^ cells/well) by using a 6-well transwell plate [49]. Briefly, cells were divided into four groups, including blank (only B16 cells), control (B16 cells co-cultured with RAW 264.7 cells), and 25 and 50 μg/mL of TFPS-treated (B16 cells co-cultured with RAW 264.7 cells) groups. After co-culturing for 24 h, B16 cells were collected and detected by Annexin V-FITC/PI apoptosis assay kit (Invitrogen, Carlsbad, CA, USA). Flowsight flow cytometry (Merck, Darmstadt, IN, USA) was used to analyze the apoptosis rate of B16 cells. Caspase-3 activity in B16 cells was performed by the Caspase 3 Activity Assay Kit (Beyotime, Shanghai, China).

### 4.9. qRT-PCR Analysis

RAW 264.7 cells in the above four groups were collected for the analysis of mRNA. Total RNA was extracted using TRIzol reagent and the RNA concentration was determined by Synergy Hi Multi-Mode reader. Then, qRT-PCR was performed by using a qTOWER3G Real-Time System (Analytik-Jena, Jena, Germany). Cycle threshold (CT) values were exported from the qPCRsoft 4.0 (Analytik Jena, Jena, Germany) and the relative abundance of RNA was calculated in each sample using the ΔΔCT method [50]. The primers used for target genes were presented in Table S1.

### 4.10. Cell Migration Assay

An in vitro scratch assay was used to assess cell migration [29]. Briefly, RAW 264.7 cells (2 × 10^6^ cells/well) were seeded at 12-well plates for 24 h. Then, cells were scratched using a pipette tip and treated with different doses of TFPS (1, 12.5, 25 μg/mL) or 1 μg/mL of LPS for 24 h. Images were captured at 0 h and 24 h, and cell migration distance was measured by using Image J (Bethesda, MD, USA).

### 4.11. Determination of Phagocytosis

The phagocytic ability of RAW 264.7 cells was measured by FITC-dextran [51]. RAW 264.7 cells (1 × 10^6^ cells per well) were seeded in a 6-well plate and incubated for 24 h. Then, cells were treated with different doses of TFPS (1, 12.5, 25 μg/mL) or 1 μg/mL of LPS for 24 h. After discarding the supernatant, the cells were incubated with a fresh medium containing FITC-dextran (1 mg/mL) at 37 °C for 1 h. The phagocytosis of FITC-dextran in RAW 264.7 cells was analyzed by Flowsight flow cytometry (Merck, Darmstadt, IN, USA).

### 4.12. Measurement of NO Production

RAW 264.7 cells (2 × 10^5^ cells/well) were incubated in 24-well plates for 24 h and were then treated with different doses of TFPS (1, 12.5, 25 μg/mL) or 1 μg/mL of LPS. After 24 h, the supernatants were collected and dispensed into a 96-well plate (50 μL/well). Then, an equal volume of Griess solution was added. The plate was incubated at room temperature for 15 min. Finally, the absorbance at 540 nm was measured by using a microplate reader.

### 4.13. Measurement of ROS

RAW 264.7 cells (2 × 10^5^ cells/well) were treated with different doses of TFPS (1, 12.5, 25 μg/mL) or LPS in 24-well plates for 24 h. After discarding the supernatant, the cells were incubated with DHFH-DA (20 μM) for 30 min. DCFH-DA solution was then discarded, and the cells were washed gently with PBS twice [52]. Finally, the cells were collected, and intracellular ROS were measured by flow cytometry (Becton-Dickinson, Franklin Lakes, NJ, USA).

### 4.14. Enzyme-Linked Immunosorbent Assay (ELISA)

The concentration of IL-6, TNF-α, and IFN-γ in RAW cells was determined by ELISA assay according to the kit instructions. After 24 h of being treated with different doses of TFPS (1, 12.5, 25 μg/mL) or LPS, the supernatants were collected for detection of IL-6, TNF-α, and IFN-γ according to the manufacturer’s instructions of the ELISA kit.

### 4.15. Western Blot Analysis

RAW 264.7 cells (5 × 10^5^ cells/mL) were seeded in a 100 mm dish. After 24 h, the cells were treated with different doses (1, 12.5, 25 μg/mL) of TFPS or LPS for 1 h. The cells were then collected, and 200 μL of ice-cold RIPA buffer was added for incubation for 10 min on ice. Each sample was centrifuged at 4 °C and 12,000 rpm for 10 min. The BCA assay was used to measure the protein concentration. An equal amount of protein (40 μg) from each sample was separated by SDS-PAGE and transferred to a 0.45 μm nitrocellulose membrane (NC, BIO-RAD, Germany). The NC membrane was blocked with 5% (*w*/*v*) BSA and incubated with primary antibodies at 4 °C overnight. The membrane was then washed 3 times with TBST for HRP-conjugated specific secondary antibody detection. ECL kits (Thermo, Rockford, IL, USA) were used to detect immunoreactive proteins.

### 4.16. Network Pharmacology Analysis

TAMs-related targets were collected by searching for the keyword (tumor-associated macrophages) from the GeneCards (www.genecards.org/, accessed on 3 April 2022) database. Potential targets of TFPS were obtained from the TCMSP database (http://tcmspw.com/tcmsp.php, accessed on 3 April 2022), HERB databases (http://herb.ac.cn, accessed on 3 April 2022), and HIT databases (http://lifecenter.sgst.cn/hit/, accessed on 3 April 2022) according to the identified component. Venny 2.1.0 (https://bioinfogp.cnb.csic.es/tools/venny/, accessed on 3 April 2022) was used to obtain intersected targets of TFPS and TAMs.

To understand associations between the intersected targets, we used STRING (https://string-db.org/, accessed on 3 April 2022) to perform network topology analysis and constructed a protein–protein interaction (PPI) network. In the network, the higher the degree of the target, the higher the number of other proteins related to it. Finally, ten targets with the highest degree were visualized using Cytoscape (version 3.9.0). The redder the color of the node, the higher the degree.

Through the DAVID (https://david-d.ncifcrf.gov/, accessed on 3 April 2022) online platform, Gene Ontology (GO) and Kyoto Encyclopedia of Genes and Genomes (KEGG) enrichment analysis of intersected targets of TFPS and TAMs were performed. The GO terms were divided into three categories, including biological process (BP), cellular component (CC), and molecular function (MF). Results were screened at *p* < 0.05. The GO terms (top 10 of BP, CC, and MF) and KEGG pathway (top 20) were plotted through an online platform (www.bioinformatics.com.cn, accessed on 3 April 2022).

### 4.17. Cell Immunofluorescence

RAW264.7 cells were seeded in a laser confocal culture dish (Corning Life Science, Lowell, CA, USA) for immunofluorescence staining. After TFPS (1, 12.5, 25 μg/mL) treatment, the cells were washed with PBS, fixed with 4% paraformaldehyde solution for 15 min, and permeabilized with 2.5 g/L Triton X-100 for 10 min. After blocking with 1% BSA (1 g BSA + 100 mL TBST) for 1 h, the cells were incubated with p65 antibody (1:400) at 4 °C overnight, followed by incubating with FITC-conjugated goat anti-rabbit IgG (1:500) for 1 h. Then, the nucleus was stained with DAPI for 10 min. Finally, images of immunofluorescence were obtained by using a high-resolution confocal laser microscope.

### 4.18. Statistical Analysis

Statistical analysis was performed using mean ± standard error (SEM) and one-way ANOVA. Graph Pad Prism 8 software (Graph Pad Prism Inc., San Diego, CA, USA) was used to analyze the experimental data, with *p* < 0.05 as the significance, and make the charts.

## 5. Conclusions

For the first time, our study demonstrated the anti-tumor mechanism of TFPS in macrophages and melanoma co-culture systems. The TFPS used in this study comprised xylose, glucose, mannose, and galactose. We found that TFPS exerts anti-tumor activity by regulating the M1 polarisation of macrophages, rather than directly targeting tumor cells. Molecular mechanism studies showed that TFPS induces activation of the MAPK and NF-κB signaling pathways. These findings help us better understand the mechanism by which TFPS enhances the M1 macrophage phenotype and induces apoptosis in B16 cells and suggest that TFPS may be applied as an immunomodulatory agent for cancer therapy.

## Data Availability

Data are contained within the article and Supplementary Materials.

## Supplementary Materials

The following supporting information can be downloaded at: https://www.mdpi.com/article/10.3390/molecules28104018/s1, Figure S1: KEGG pathway. (**A**,**B**) Part of the IL-17 signaling pathway and TNF signaling pathway obtained from the Kyoto Encyclopedia of Genes and Genomes (KEGG, https://www.kegg.jp/, accessed on 21 September 2021); Table S1: Primers used for qRT-PCR.

## Sample Availability

Samples of the compounds TFPS are available from the authors.
