# Supplementary material for: Tremella fuciformis Polysaccharide Induces Apoptosis of B16 Melanoma Cells via Promoting the M1 Polarization of Macrophages
Source: Molecules. 2023 May 11;28(10):4018. doi: 10.3390/molecules28104018 (PMC10220659; doi:10.3390/molecules28104018)
Supplement: Supplementary file 1 [file molecules-28-04018-s001.zip › molecules-2331117-supplementary/Table S1.pdf]

Supplementary Table S1. Primers used for qRT-PCR

Gene	Direction	Sequence
Gapdh	forward	5'-AACTTTGGCATTGTGGAAGG-3'
Gapdh	Reverse	5'-ACACATTGGGGGTAGGAACA-3'
iNOS	forward	5'-AACGGAGAACGTTGGATTTG-3'
iNOS	Reverse	5'-CAGCACAAGGGGTTTTCTTC-3'
Arg1	forward	5'-GCTGTCTTCCCAAGAGTTGGG-3'
Arg1	Reverse	5'-ATGGAAGAGACCTTCAGCTAC-3'
CD80	forward	ACCCCCAACATAACTGAGTCT
CD80	Reverse	TTCCAACCAAGAGAAGCGAGG
CD260	forward	CTCTGTTCAGCTATTGGACGC
CD260	Reverse	CGGAATTTCTGGGATTCAGCTT
