# Supplementary material for: Tremella fuciformis Polysaccharide Induces Apoptosis of B16 Melanoma Cells via Promoting the M1 Polarization of Macrophages
Source: Molecules. 2023 May 11;28(10):4018. doi: 10.3390/molecules28104018 (PMC10220659; doi:10.3390/molecules28104018)
Supplement: Supplementary file 1 [file Figure S1.pdf › molecules-2331117-supplementary/Figure S1.pdf]

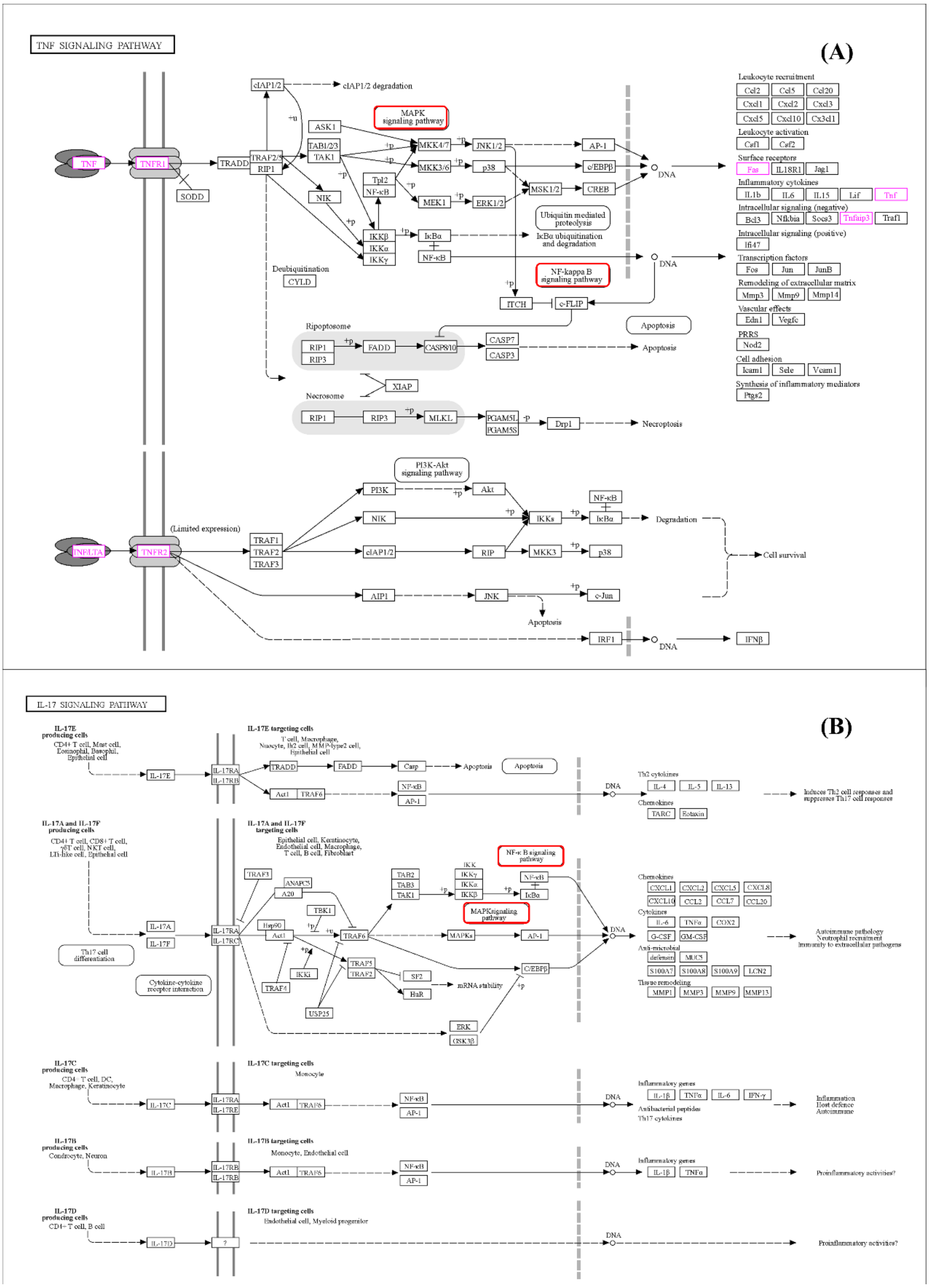

Figure S1. KEGG pathway. (A-B) Part of IL-17 signaling pathway and TNF signaling pathway obtained from Kyoto Encyclopedia of Genes and Genomes (KEGG, https://www.kegg.jp/, accessed on 21 September 2021).
